# Segmentation of a Vibro-Shock Cantilever-Type Piezoelectric Energy Harvester Operating in Higher Transverse Vibration Modes

**DOI:** 10.3390/s16010011

**Published:** 2015-12-23

**Authors:** Darius Zizys, Rimvydas Gaidys, Rolanas Dauksevicius, Vytautas Ostasevicius, Vytautas Daniulaitis

**Affiliations:** 1 Faculty of Mechanical Engineering and Design, Kaunas University of Technology, Studentu 56, Kaunas LT-51368, Lithuania; rimvydas.gaidys@ktu.lt; 2 Institute of Mechatronics, Kaunas University of Technology, Studentu 56-123, Kaunas LT-51368, Lithuania; rolanas.dauksevicius@ktu.lt (R.D.); vytautas.ostasevicius@ktu.lt (V.O.); 3 Faculty of Informatics, Kaunas University of Technology, Studentu 50, Kaunas LT-51368, Lithuania; vytautas.daniulaitis@ktu.lt

**Keywords:** piezoelectric, optimal segmentation, vibration energy harvesting, resonant frequency, strain node, numerical modelling

## Abstract

The piezoelectric transduction mechanism is a common vibration-to-electric energy harvesting approach. Piezoelectric energy harvesters are typically mounted on a vibrating host structure, whereby alternating voltage output is generated by a dynamic strain field. A design target in this case is to match the natural frequency of the harvester to the ambient excitation frequency for the device to operate in resonance mode, thus significantly increasing vibration amplitudes and, as a result, energy output. Other fundamental vibration modes have strain nodes, where the dynamic strain field changes sign in the direction of the cantilever length. The paper reports on a dimensionless numerical transient analysis of a cantilever of a constant cross-section and an optimally-shaped cantilever with the objective to accurately predict the position of a strain node. Total effective strain produced by both cantilevers segmented at the strain node is calculated via transient analysis and compared to the strain output produced by the cantilevers segmented at strain nodes obtained from modal analysis, demonstrating a 7% increase in energy output. Theoretical results were experimentally verified by using open-circuit voltage values measured for the cantilevers segmented at optimal and suboptimal segmentation lines.

## 1. Introduction

Various different approaches exist for vibration energy harvesting via piezoelectric, electromagnetic and electrostatic transduction mechanisms, which have been widely discussed and compared by [1,2,3]. The piezoelectric transduction mechanism was extensively studied in recent years [3] and proven to be a prime choice for MEMS energy harvesting from harmonic ambient vibrations [4]. A piezoelectric energy harvester usually constitutes a cantilevered transducer with single or multiple layers of piezoelectric material bonded on its surface. The transducer is mounted on a vibrating structure for voltage generation via a direct piezoelectric effect. Vibration-based energy harvesters are usually designed to exhibit natural frequencies that match ambient vibration frequencies. Various authors have focused on modeling mechanical [5] and electronical [6] aspects of piezoelectric energy harvesting, as well as on different optimization techniques to increase the efficiency of the electromechanical conversion [7,8,9,10]. The qualitative factors of a vibration energy harvesting system have been recognized by [2,11]. A narrow bandwidth is a major issue for transducers with a high quality factor, and this issue has been addressed by the authors of [12], who developed a design of plate structures for vibration energy harvesting from two or more modes of vibration to respond to variable frequency sources of base excitation. Different approaches to multi-modal harvesters were further investigated by [13,14].

Mechanical-to-electrical energy conversion can be investigated based on piezoelectric constitutive laws [4] and fundamental relations of mechanics of materials [15]. The electric charge collected at the electrodes is the integral of the normal component of electric displacement over the electrode area, and the electric displacement field generated in the piezoelectric layer during vibration is a function of the strain distribution over transducer length. If the strain distribution and the corresponding electric displacement component changes sign under full-sheet electrodes, a cancellation effect manifests, leading to a substantial reduction in piezoelectric charge output [16,17,18]. In higher vibration modes (second and above), a certain strain node is present, where the strain field changes sign, meaning that if a continuous electrode is applied on the surface, a significant cancelation in collected charge occurs, resulting in losses in harvested energy. Several works were published investigating the normal strain nodes in higher modes [19] and proper segmentation techniques [16,17] for the maximization of harvested energy. Besides the conventional vibration energy harvesting from harmonic vibrations in resonant or off-resonant mode, there were a series of harvesters dedicated to impact energy harvesting [20,21]. Modeling of contact dynamics aspects was thoroughly investigated in [22].

During an impact, not only the first natural vibration mode is excited, but also higher natural modes, so the cantilever shape after the impact may be represented as a superposition of the first and higher natural modes. The number of modes will participate in cantilever vibration as separate components of periodic vibration [23]. Therefore, in practice, higher modes of the harvester can be excited due to the random, varying frequency or impulse-type excitations generated by ambient vibration sources [17]. Bearing in mind the latter, [24] focused on the dynamic efficiency of the cantilever vibrating in its third natural mode. The authors proposed a few approaches of the excitation of the third natural mode, namely vibro-impact or forced excitation. In [16,17,24], the need for proper segmentation at the strain nodes of the cantilevers vibrating in higher natural modes was highlighted. The authors of [10,25] proposed a multi-beam piezoelectric energy harvester that exploits impact to transform low-frequency ambient mechanical vibrations toward higher resonant frequencies of the piezoelectric transducers. The system consists of a steel driving beam that is exciting two piezoelectric beams via impact. The authors of [26] proposed a mechanism for achieving frequency up-conversion for low frequency harvesters exploiting impact between end-stop and a cantilever beam: a seven-fold increase in the oscillation frequency of the transducer was induced if compared to the base excitation frequency.

In this paper, the ideas presented by [16,17,19] are further developed. The positions of strain nodes of a cantilevered Euler–Bernoulli beam without a tip mass for second natural frequency is calculated from modal analysis and compared to the strain node found from transient analysis. During the transitional processes, higher transient vibration modes are excited in vibro-impact harvesters. Therefore, in contrast to the conventional piezoelectric vibration energy harvester (PVEH), the piezoelectric layer has to be segmented to maximize the energy output and avoid the generated charge cancelation due to effects related to strain nodes. Two setups of segmentation were investigated in this work: one obtained from modal analysis (in further sections, referred to as suboptimal) and the other from transient analysis, further referred to as optimal segmentation.

## 2. FEM Modelling of PVEH Segmentation in Higher Vibration Modes

Two types of cantilevers were chosen for investigation: a conventional cantilever of a constant cross-sectional area and a cantilever of optimized shape. The latter refers to a cantilever that has a minimal volume and the same second transverse vibration Eigen frequency $\omega_{2}$ as the cantilever of a constant cross-section. The procedure for cantilever shape optimization is given in further sections. Both setups had a pair of PVDF (polyvinylidene fluoride) layers attached on their top surface. Cantilevers were modeled as uniform composite beams for linearly elastic deformations and geometrically small oscillations based on the Euler–Bernoulli beam assumption.

**Figure 1:**
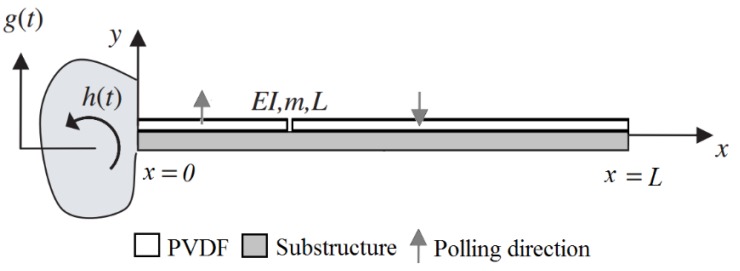
Piezoelectric cantilever under translational and small rotational base motions (adapted from [17]). PVDF, polyvinylidene fluoride.

The effects of shear deformations and rotary inertia are neglected, and this is a reasonable assumption, since typical piezoelectric cantilevers are designed and manufactured as thin beams, as described in [6]. The principal scheme of the cantilever of a constant cross-section is provided in Figure 1. The mechanical properties and dimensions of both cantilevers are listed in Table 1. The first PVDF layer is mounted from the fixed end to the strain node, the second from the strain node up to the cantilever end. The properties of the PVDF are given in Table 2. The Eigen frequencies of both cantilevers were obtained from the modal analysis.

**Table 1:** Mechanical and geometrical properties of the considered cantilever setups.

Parameter	Constant Cross-Section Area Cantilever	Optimal Shape
$\omega_{1},\;\text{Hz}$	86	66
$\omega_{2},\text{ Hz}$	541	534
Density, kg/m^3^	7850	
Elastic modulus, N/m^2^	2 × $10^{10}$	
Poisson’s ratio	0.33	
Length, m	0.1	
Width *a*, m	0.01	
Thickness *b*, m	1 × 10^−3^	Varying from 4 × 10^−4^ to 1 × 10^−3^

**Table 2:** PVDF properties.

Parameter	Name	PVDF	Units
d_31_	Piezoelectric strain constant	23	(pC/N)
g_31_	Piezoelectric stress constant	216	(10^−3^ Vm/N)
k_t_	Electromechanical coupling factor	12%	
C	Capacitance	1.4–2.8	nF
Y	Young’s modulus	4	10^9^ N/m^2^
ε	Permittivity	110	10^−12^ F/m
ρ	Mass Density	1780	kg/m^3^
t	Thickness	64	µm

### 2.1. Finite Element Model

The differential equations were solved numerically using the finite element method. The cantilevers under investigation are considered to be thin, which is important, because in thin beams, the shear deformations in the transverse direction are neglected. The PVEH model is composed of two piezoelectric layers of opposite polarities, covered on both sides by electrode layers, mounted on top of a substructure and connected in a series configuration. The function of the electrode is to provide uniform potential. Electromechanical modeling of cantilever-type piezoelectric transducers has been studied by several authors in the past [4,5]. The cantilever was excited by harmonic base movement with frequencies matching the second natural frequency of the cantilevers (see Table 1) with an acceleration of 1.3 g. Equation (8) was used for modal analysis, while Equation (13) was used to solve the transient analysis problem. Excitation parameters are varied by the frequency ω_n_ of the time-dependent force *f(t)*. A 2D in-plane strain application was applied for modeling. The element type that was used is a quadratic Lagrange element with second-order polynomial approximation. FE mesh with 350 elements per length of the cantilever was implemented. The Structural Mechanics module of COMSOL was adopted for the calculation of the Eigen frequencies and the strain nodes of the cantilevers. Simulation results were obtained by deriving voltage output from piezo plane strain analysis. The study was performed both for the cantilever of a constant cross-section and the optimally-shaped cantilever (Table 1), which was obtained by solving the shape optimization problem with the objective to obtain the cantilever of minimal volume for a fixed second natural frequency of transverse vibrations.

### 2.2. Constitutive Equations for Substructure and Piezoelectric Layers

The structure is composed of a load-bearing material and two layers of piezoelectric material. Under the linear elasticity assumption, the constitutive equation for the load-bearing material is given as Equation (1):

(1) $$T=C^{H}S$$

where *T* is mechanical stress, $C^{H}$ is the elasticity matrix of the host layer and *S* is strain. The electromechanical coupling effect of the piezoelectric material can be described by the following constitutive Equations (2) and (3).

(2) $$T=C^{P}S-eE\;S$$

(3) $$D=e^{T}S+\varepsilon^{S}E\;S$$

where $C^{P}$ is the elasticity matrix of the piezoelectric layer, *D* denotes the electric displacement, e and *E* are the piezoelectricity matrix and applied electric field, respectively, and $\varepsilon^{\text{S}}$ is the permittivity matrix. Constitutive equations are further described by [27]. It can be assumed that the electric potential varies linearly across the thickness of the piezoelectric layer. The electrical boundary condition is open circuit, thus D=0. If an axial stress *T_1_* is applied, it will deform, and hence, the charge will displace toward the electrodes. Under open circuit conditions (*D = 0*), the voltage *V* is given by:

(4) $$V=g_{31}hT_{1}$$

As the substrate vibrates in its second natural frequency, the PVDF layers undergo dynamic strain, thus generating alternating voltage output via direct piezoelectric effect.

### 2.3. Procedure for Cantilever Shape Optimization

The optimal shape of the cantilever was obtained by subjecting it to the optimization procedure as follows: minimize the volume of the cantilever-type harvester.

(5) Min Volume (x)

defined by state equation:

(6) $$K\left(x\right)v=\omega_{i}^{2}M\left(x\right)v$$

Here, $\omega_{i}$ is the natural frequency and v is the “mode shape” of the system. With a constraint of the shape:

(7) $$x_{min}<x<x_{max}$$

and Eigen frequency:

(8) $$w=w^{*}$$

**Figure 2:**
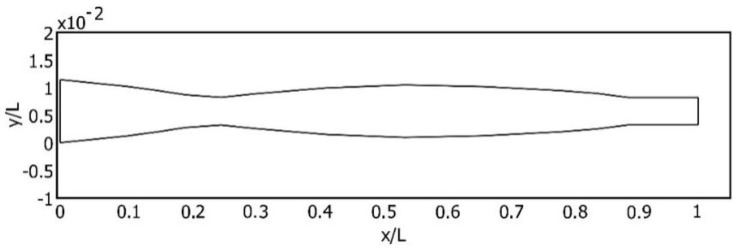
Normalized shape of the optimally-shaped cantilever.

Here, *x* is the vector of the design parameters, w the Eigen frequency of the cantilever and $w^{*}$ the desired frequency. The application of this optimization procedure resulted in the optimal shape of the cantilever for the second transverse vibration (Figure 2).

### 2.4. Determination of Strain Node Position for the Second Vibration Mode via Modal Analysis

For modal analysis, the cantilever is considered undamped and undergoing free vibrations; thus, the governing equation as following.

(9) $$M\ddot{u}+Ku=0$$

Internal elastic forces Ku act as an offset to the internal forces $M\ddot{u}$. In this case, *K* is the stiffness matrix, and *M* is the mass matrix. Reducing Equation (9), assuming the solution of Equation (10) can be derived and Eigen frequencies of the cantilever obtained.

(10) $$Kv=\omega_{i}^{2}Mv$$

One end of the cantilever is fixed and the second end is free, as shown in Equation (11) for the fixed end and Equation (12) for the free end, where Y(x) is displacement in the y direction at distance x from the fixed end.

(11) $$x=0,\;Y\left(x\right)=0,\;\frac{dY\left(x\right)}{dx}=0$$

(12) $$\text{x}=L,\;\frac{d^{2}Y\left(x\right)}{dx^{2}}=0,\;\frac{d^{3}Y\left(x\right)}{dx^{3}}=0$$

**Figure 3:**
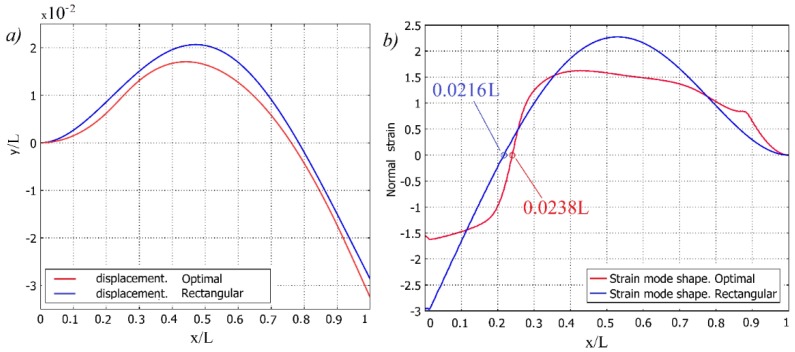
Second transverse vibration mode of a cantilever: (**a**) displacement; (**b**) normal strain.

**Figure 4:**
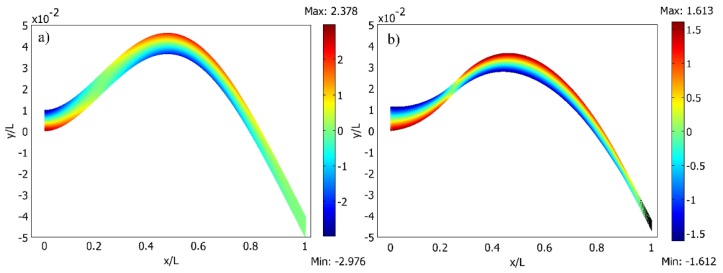
The second transverse vibration mode of the cantilever and the field of normal strain distribution: (**a**) cantilever of a constant cross-section; (**b**) optimally-shaped cantilever.

Figure 3a shows the second transverse vibration mode shape of both cantilevers obtained by modal analysis. Meanwhile, Figure 3b illustrates the strain mode shape for the second natural mode of the cantilever of the constant cross section and optimally shaped cantilever; rough estimation of strain node locations is 0.216 L and 0.238 L, respectively. The location of strain node of a cantilever of constant cross section complies well with [17] that predicted a strain node of second natural mode to be at 0.2165 L. The slope of strain curve of optimally shaped cantilever in region of strain node is higher. This means that interval of cantilever length with different sign strain is shorter. Figure 4 shows the second normal mode shapes of both cantilevers with normal strain distribution along their faces for cantilever of constant cross section and optimally shaped cantilever in Figure 4a,b, respectively.

It can be observed that the upper and lower faces of the cantilever are subjected to strain of different signs; while the upper face is negatively strained (compressed) at x/L = 0, the lower face is under tension.

Strain distribution over the length of the beam changes sign, and collecting the induced piezoelectric charge with continuous electrodes results in an electrical cancellation effect accompanied by a reduction in harvested energy.

### 2.5. Determination of Strain Node Position for the Second Vibration Mode via Transient Analysis

The transient analysis of the cantilever was conducted to verify the position of the strain node in the cantilever during its base excitation with time varying force *f(t)* acting on it. The corresponding equation of motion is given in Equation (13).

(13) $$M\ddot{u}\left(t\right)+C\dot{u}\left(t\right)+Ku\left(t\right)=f\left(t\right)$$

Here, *C* is the damping matrix. The time-dependent force f(t) is described as cantilever body load in the vertical direction and is defined as force/volume using the thickness.

(14) $$f\left(t\right)=am\;sin\;\omega_{n}t$$

where a is acceleration, m is the mass of the system and $\omega_{n}$ is the excitation frequency. The equation of motion controls the linear dynamic behavior, and the dynamic response can be found by solving this equation of motion.

**Figure 5:**
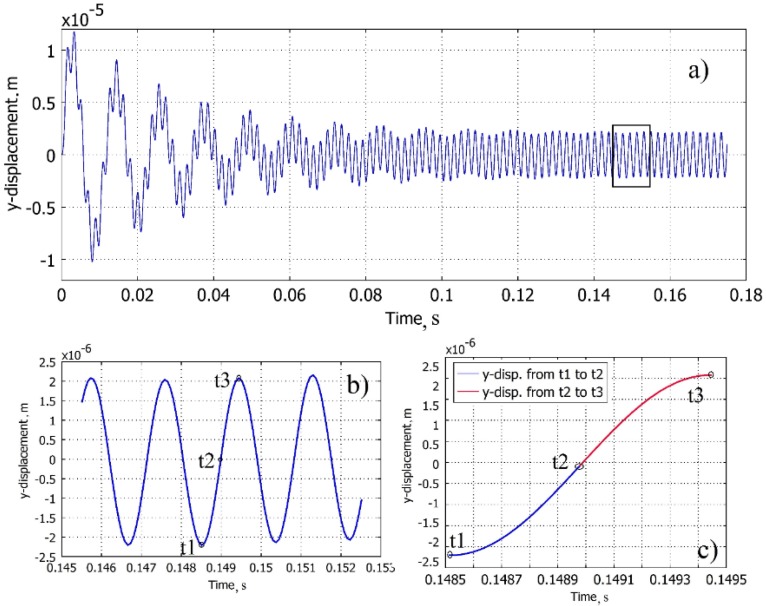
Cantilever free end displacement in the transverse direction: (**a**) dynamical process; (**b**) region of steady-state vibrations; (**c**) ½ T period of vibration for the analysis of the position of the cantilever strain nodal point.

The transverse displacement of the free end of the cantilever for a given interval of time can be seen in Figure 5a. Figure 5b indicates that the period selected for further investigation is at the region of steady-state vibrations. Half of the period is selected for the investigation of the normal strain distribution along the face of the cantilever, as illustrated in Figure 5c.

The first quarter of period (*t_1_, t_2_*) is colored in blue and the second (*t_2_, t_3_*) in red for the clarity of the transition processes taking place in the face of the cantilever during the vibration.

**Figure 6:**
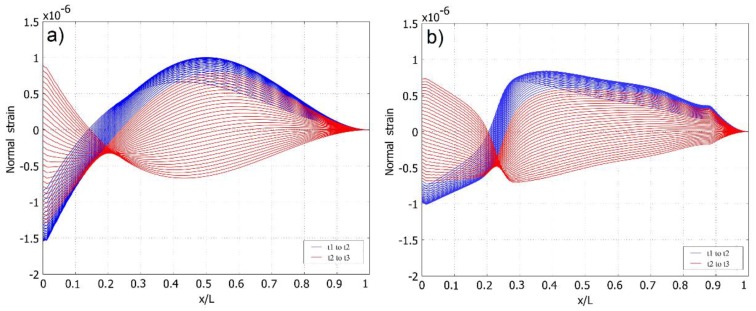
Normal strain distribution *versus* the length of the cantilever per ½ T obtained from transient analysis: (**a**) cantilever of a constant cross-section; (**b**) optimally-shaped cantilever.

**Figure 7:**
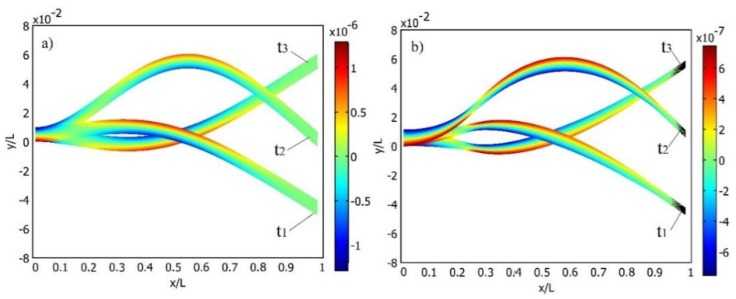
Mode shape and total normal strain distribution of the constant cross-section area cantilever at t_1_, t_2_ and t_3_: (**a**) cantilever of a constant cross-section; (**b**) optimally-shaped cantilever.

**Figure 8:**
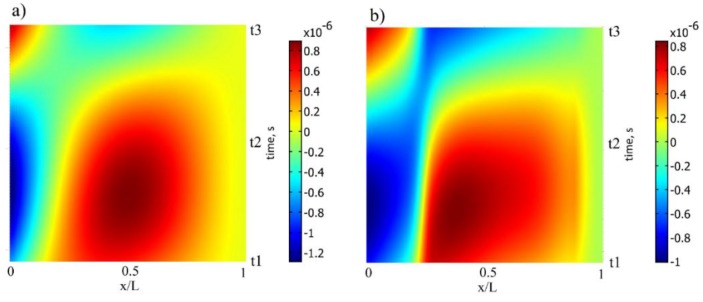
Normal strain distribution on the upper face of the cantilever in ½ T: (**a**) cantilever of a constant cross-section; (**b**) optimally-shaped cantilever.

The same coloring scheme is used in Figure 6, where the normal strain distribution along the upper face of both cantilever setups is shown per ½ T of the cantilevers’ transverse vibration. The number of curves in Figure 6 represent the number of interpolated time step values $\Delta t_{i}$ between *t_1_* and *t_3_*. Figure 6 and Figure 7 clearly reveal that, overall, normal strain output amplitudes are higher in the case of the cantilever of a constant cross-section. Meanwhile, the normal strain mode shape of the optimally-shaped cantilever forms a steeper angle at the cantilever end and is close to the predicted strain node, thus a higher strain density can be predicted. This forms strain mode curves that are stable at high strain output zones. In Figure 8, strain distribution along the face of the cantilever (Figure 8a: cantilever of a constant cross-section; Figure 8b: optimally-shaped cantilever) *versus* time can be observed.

The horizontal axis of Figure 8 represents a normal strain distribution along the upper face of the cantilever of a constant cross-section at time instant $t_{i}$, while the vertical axis represents strain change at any given point per time interval [*t_1_, t_3_*]. It can be observed that the cantilever of a constant cross-section produces higher strain amplitudes though the optimally-shaped cantilever exhibits larger average strain output with higher strain mode slopes.

Numerical analysis was performed in MATLAB in order to post-process results obtained from the COMSOL environment. Figure 6a,b demonstrates that the strain node is not as exactly defined; thus, a methodology has been developed to determine the strain node from transient analysis and to compare the amount of normal strain output of the cantilever for both. As the cantilever is vibrating, at any interpolated time step $\Delta t_{i}$, the cantilever is experiencing both compression and tension at the same face; this produces a drifting strain node where the normal strain is equal to zero. $\Delta t_{i}$ is calculated as shown in Equation (15), where *N* is the number of integration steps.

(15) $$\Delta t=\frac{t_{3}-t_{1}}{N}$$

**Figure 9:**
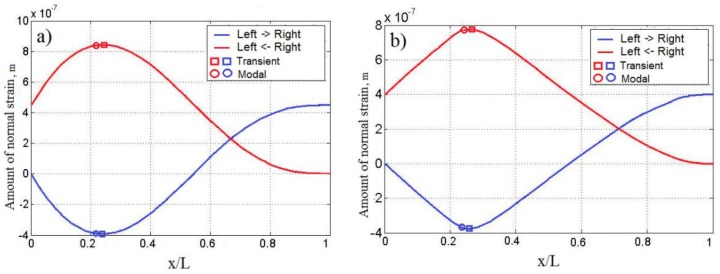
Accumulation of normal strain over length x/L per ½ *T*: (**a**) cantilever of a constant cross-section; (**b**) optimally-shaped cantilever. The blue curve represents the integration from the fixed end to the free end of the cantilever, red the integration of the opposite direction.

The aim of this optimization problem is to determine the exact strain node position along the cantilever by using transient analysis, where the average strain output per ½ T is equal to zero. The strain node from the transient analysis is estimated by integrating the area bounded by each interpolated time step (or curve) over an increment of the length of the cantilever L. The Simpson method was used for integration; the approximation is given in Equation (16).

(16) $$\int_{0}^{L}\frac{du}{dx}dx\approx \frac{h}{3}\left[\frac{d\left(u_{0}\right)}{dx}+2\overset{\frac{N}{2}-1}{\underset{j=1}{\sum }}\frac{d\left(u_{2j}\right)}{dx}+4\overset{N/2}{\underset{j=1}{\sum }}\frac{d\left(u_{2j-1}\right)}{dx}+\frac{d\left(u_{N}\right)}{dx}\right]$$

where u is displacement in the axial direction along the cantilever. Numerical results obtained from COMSOL simulations were exported to MATLAB for further post-processing. To calculate the amount of normal strain at each time step $\Delta t_{i}$, the normal strain curve integration along the length of the cantilever was performed from both ends of the cantilever, as depicted in Figure 9a,b for a cantilever of a constant cross-section and an optimally-shaped cantilever, respectively. The blue curve represents the integration from the fixed end to the free end, red the integration of the opposite direction.

The curves represent the accumulation of the normal strain over an increment of length L of the cantilevers. Total strain per ½ *T* is negative at the fixed end and increases until it reaches the maximum at 0.216 L. The square marker in Figure 9a denotes the calculated maximum of the curve with the strain node obtained from the transient analysis, while the round marker denotes the normal strain output at the strain node obtained from the modal analysis (from Figure 3b).

**Table 3:** Comparison of normal strain amount.

	Strain Node	Amount of Normal Strain-Left (Dimensionless)	Amount of Normal Strain-Right (Dimensionless)	Gain, %
Cantilever of a constant cross-section				
Modal solution	0.216	6.53 × 10^−10^	−9.7 × 10^−10^	
Transient solution	0.238	6.73 × 10^−10^	−9.9 × 10^−10^	+5.5%
Optimally-shaped cantilever				
Modal solution	0.239	6.3 × 10^−10^	−9.6 × 10^−10^	
Transient solution	0.259	6.5 × 10^−10^	−9.85 × 10^−10^	+5.2%

Table 3 summarizes the comparison of the effective total normal strain output integral for cantilevers segmented in strain node, determined via modal analysis, and a strain node obtained from the transient analysis. The total effective strain output consists of two components: strain produced by the region to the left of the strain node and the region to the right of the strain node, as illustrated in Figure 9a for the cantilever of a constant cross-section and Figure 9b for the optimally-shaped cantilever. Table 3 indicates that per ½ *T*, the left side of the cantilever of a constant cross-section (representing the first segment of the harvester) segmented at the strain node obtained from the modal solution undergoes strain equal to 6.53 × 10^−10^, while the cantilever segmented at the strain node obtained from the transient solution underwent 6.73 × 10^−10^, which is equal to a 3% increase in strain output. The right-hand side (representing the second segment) underwent 2.5% more strain; thus, the total gain of the transient *versus* modal solution was 5.5% more effective strain per ½ *T* of the second transverse vibration mode. Accordingly, the optimally-shaped cantilever segmented at the strain node obtained from the transient solution produced 5.2% more strain if compared to the strain output from a cantilever segmented at the strain node obtained from modal analysis.

**Figure 10:**
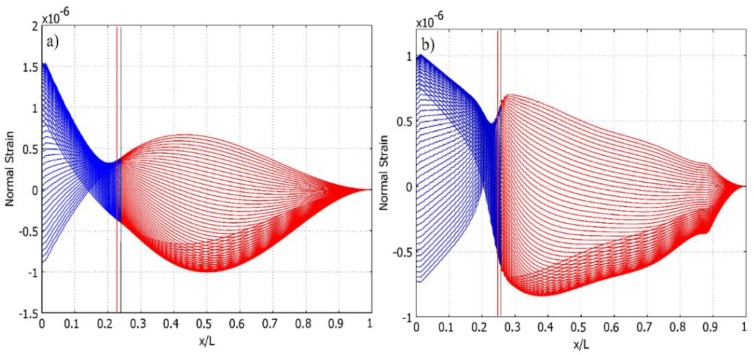
Normal strain distribution *vs*. the length of the cantilever per ½ T from transient analysis and the segmentation nodal point: (**a**) cantilever of a constant cross-section; (**b**) optimally-shaped cantilever. Vertical red line: nodal point position determined from modal analysis; black: from transient analysis.

Figure 10 illustrates the importance of the correct selection of the strain node for the segmentation of a piezoelectric cantilever subjected to ambient harmonic excitation that matches the second natural frequency of transverse vibrations of the cantilever. The red and black vertical lines in Figure 10 represent the location of segmentation lines at strain nodes obtained from modal and transient analysis, respectively. Table 3 indicates that the difference between two lines is 2.2 × 10^−2^ L for the cantilever of a constant cross-section and 2 × 10^−2^ L—for the optimally-shaped cantilever. Transferal of the segmentation line to the strain node obtained from transient analysis produced 5.5% and 5.2% more strain from the cantilever of a constant cross-section and the optimally-shaped cantilever, respectively, if compared to the modal solution. The period of vibration was chosen as ½ *T* because full transition from maximum negative to maximum positive strain output values can be observed.

## 3. Theoretical and Experimental Results: Open Circuit Voltage Outputs

**Figure 11:**
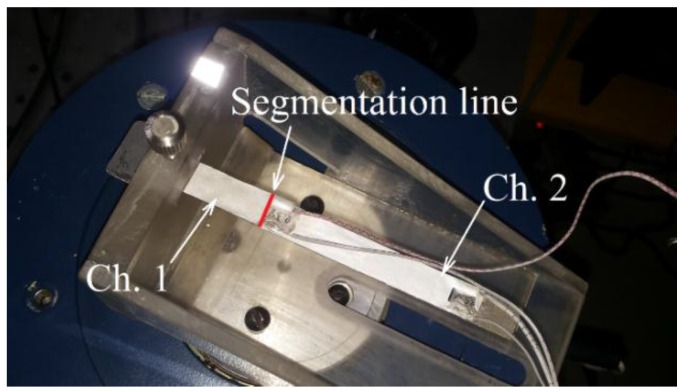
Experimental setup of the tested piezoelectric vibration energy harvester.

**Figure 12:**
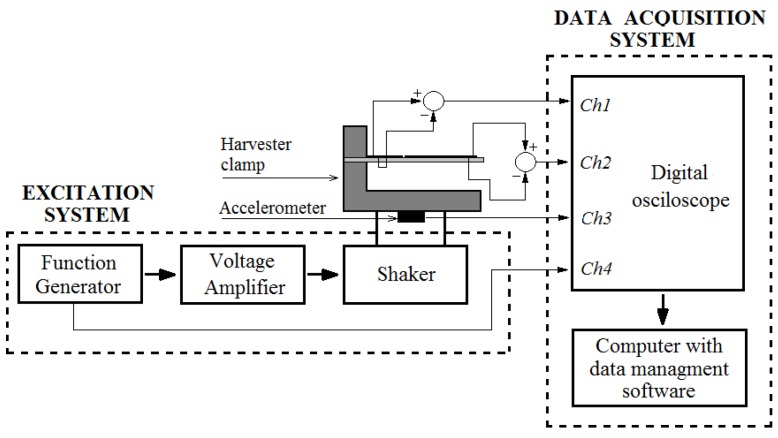
Scheme of the experimental setup.

Further, open-circuit voltage output generated by the cantilever of a constant cross-section and the optimally-shaped cantilever was compared to the experimentally-obtained open-circuit voltage values. The experimental setup and scheme are illustrated in Figure 11 and Figure 12.

**Figure 13:**
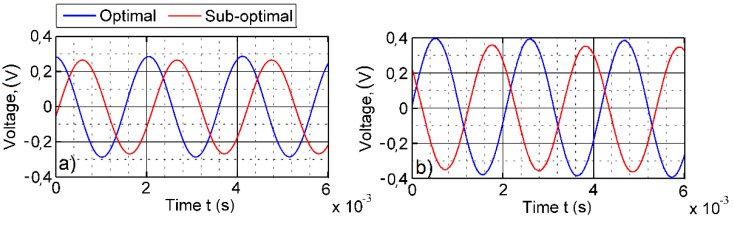
Open circuit voltage output for the cantilever of a constant cross-section ($\omega_{2}$ = 551 Hz): (**a**) first segment; (**b**) second segment.

The experimental setup shown in Figure 12 consists of a piezoelectric vibration energy harvester and two systems connected to it, the excitation system and the data acquisition system. The harvester clamp is mechanically mounted onto an electromagnetic shaker, which excites the PVEH. The substrate layer of the PVEH (with dimensions of 100 × 10 × 1 mm) is made of structural steel (Table 1), and two PVDF layers (transducers DT1-028K by Measurement Specialties Inc., Hampton, VA, United States) are mounted on top. The substrate layer was fabricated from structural steel by using water jet cutting. Function generator AGILENT 33220A is used to control the harmonic excitation signal transmitted to the electromagnetic shaker. Single-axis miniature piezoelectric charge-mode accelerometer METRA KS-93 (with a sensitivity of *k* = 5 mV/(m/s^2^)) is attached at the bottom of the electromagnetic shaker for acceleration measurements. The experiments were performed with constant 1.3 g acceleration. Figure 13 provides the measured open-circuit voltage outputs for the cantilever of a constant cross-section that was excited at the second natural frequency of 551 Hz.

**Figure 14:**
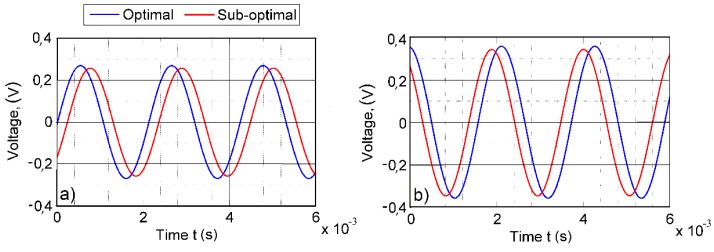
Open-circuit voltage output of the optimally-shaped cantilever at $\omega_{2}$ = 545 Hz: (**a**) first segment; (**b**) second segment.

Figure 14 presents the open-circuit output of the left-hand side electrode (Channel 1 from Figure 11) and the right-hand side electrode (Channel 2 from Figure 11). The blue and red curves represent the voltage output of the optimal and suboptimal setups, respectively. The average voltage output at Channel 1 is 0.287 V for optimal segmentation and 0.264 V for the suboptimal segmentation (8.7% difference). Channel 2 produced 0.389 V for the optimal segmentation and 0.351 V for the suboptimal segmentation (10.2% difference).

Figure 14 provides the corresponding results for the optimally-shaped cantilever excited at its second natural frequency of 545 Hz. The average voltage output at Channel 1 is 0.275 V for the optimal segmentation and 0.261 V for the suboptimal segmentation (5.1% difference). Channel 2 produced 0.369 V for the optimal segmentation and 0.353 V for the suboptimal segmentation (4.6% difference). It can be observed that the optimally-shaped cantilever produces slightly lower voltage output than the cantilever of a constant cross-section, and the optimized segmentation produces higher voltage output in all cases.

**Figure 15:**
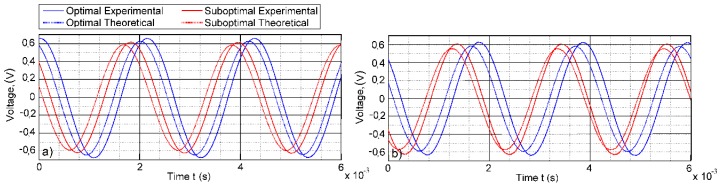
Experimental and theoretical outputs of total open-circuit voltage (first segment + second segment): (**a**) cantilever of a constant cross-section; (**b**) optimally-shaped cantilever.

These results comply with the theoretical calculations of the normal strain distribution presented in the previous section and total voltage output plots. Figure 15 provides a comparison of the theoretical and experimental total voltage outputs for the cantilever of a constant cross-section and the optimally-shaped cantilever. In every case, the theoretical predictions for the voltage output has good agreement with the experimentally-obtained voltage values. The results demonstrate that the largest voltage output was reached by the optimal setup of the cantilever of a constant cross-section (segmentation line at 0.0238 m): 0.676 V. The suboptimal setup (segmentation line at 0.022 m) of the same cantilever produced 0.615 V. The optimally-shaped cantilever with optimal segmentation (segmentation line at 0.026 m) produced 0.536 V, while the suboptimal segmentation (segmentation line at 0.024 m) 0.511 V.

## 4. Conclusions

For vibro-shock energy harvesters vibrating in higher modes, the segmentation of piezo-active layers is required due to the presence of strain nodes. Segmentation of the piezoelectric layers at the strain node obtained from modal analysis does not necessary guarantee the highest energy output. The paper presented the methodology for determining the optimal segmentation points of the piezoelectric layer vibrating in its second transverse mode.

1. The optimal segmentation point was determined by means of transient analysis for the two setups of the harvesters, the cantilever of a constant cross-section and the optimally-shaped, cantilever by integrating the strain distribution along the face of the cantilever during the ½ T period and then comparing to the total normal strain amount obtained from the cantilevers segmented at the strain node obtained from modal analysis.
2. Theoretically-obtained results indicating the superiority of the transient analysis *versus* modal analysis for finding the strain node for segmentation were verified experimentally.
3. The adjusted segmentation line increased the generated open-circuit voltage output per period of vibration by 7.2% for the optimally-shaped cantilever and 6% for the cantilever of a constant cross-section.
